# State Registration: The Central Committee and the College of Nursing

**Published:** 1916-11-18

**Authors:** 


### THE MATRONS' AND SISTERS' DEPARTMENT.

### STATE REGISTRATION.

### THE CENTRAL COMMITTEE AND THE COLLEGE OF NURSING.

Our readers, who have already learnt that negotiations have been broken off between the Central Committee for the State Registration of Nurses and the College of Nursing, will, we think, be interested in the following correspondence which has been sent us for publication by the Hon. Arthur Stanley, the Chairman of the College.

It will be remembered that from the initiation of the College Mr. Stanley made it his object, if in any way possible, to come to terms with Mrs. Bedford Fenwick, so as to go to Parliament with an agreed Bill for the State recognition of the nursing profession, the fact being recognised on both sides that a contentious measure would have no chance of passing into law in the present state of public business.

With this end in view various formal and informal conferences took place, some of which have already been reported; and we now take up the narrative with the letter from Mrs. Bedford Fenwick, dated July 17, which enclosed a memorandum headed "Bill as amended Thursday, July 13, 1916, by the Central Committee for State Registration." The letter and the memorandum have been combined in the following under the head of "The Central Committee's Remarks," so as to make clear the points raised in the letter. Throughout these Remarks we have added notes to explain what the College has done to meet the views of the Central Committee.

Those who follow this somewhat lengthy correspondence will probably come to the conclusion that Mr. Stanley went very far indeed in his endeavours to conciliate the opposition of the Central Committee, and it may well be that he is fortunate in now finding himself released from his promise to give that Committee as large a representation on the first General Council of Nursing as he was claiming for the College with its immensely more influential backing amongst the nurse-training schools and the profession at large. As we said in our issue of November 4, we hold that Mr. Stanley is absolutely right in insisting on setting out definitely in the Bill the names of the persons to be entrusted with the supremely important duty of setting up the machinery of State registration. The withdrawal of the Central Committee from the negotiations may facilitate the adhesion of some who have hitherto held aloof from registration under such auspices.

